# Rcor2 underexpression in senescent mice: a target for inflammaging?

**DOI:** 10.1186/1742-2094-11-126

**Published:** 2014-07-23

**Authors:** María J Alvarez-López, Patricia Molina-Martínez, Marco Castro-Freire, Marta Cosín-Tomás, Rosa Cristòfol, Marcelina Párrizas, Rosa María Escorihuela, Merce Pallàs, Coral Sanfeliu, Perla Kaliman

**Affiliations:** 1 Institut d’Investigacions Biomèdiques de Barcelona (IIBB), CSIC, Rosselló 161, E-08036 Barcelona, Spain; 2 Unidad de Farmacología y Farmacognósia. Institut de Biomedicina (IBUB), Universidad de Barcelona y CIBERNED. Facultad de Farmacia, Av Diagonal, 643, E-08028 Barcelona, Spain; 3 Instituto de Investigaciones Biomédicas August Pi i Sunyer (IDIBAPS), Rosellón 149, E-08036 Barcelona, Spain; 4 Instituto de Neurociencias, Departamento de Psiquiatría y Medicina Legal. Facultad de Medicina, Universitat Autònoma de Barcelona, Campus de Bellaterra, Bellaterra (Cerdanyola del Vallès), E-08193 Barcelona, Spain

**Keywords:** RCOR2, Aging, SAMP8, Inflammation, IL6

## Abstract

**Background:**

Aging is characterized by a low-grade systemic inflammation that contributes to the pathogenesis of neurodegenerative disorders such as Alzheimer’s disease (AD). However, little knowledge is currently available on the molecular processes leading to chronic neuroinflammation. In this context, recent studies have described the role of chromatin regulators in inflammation and longevity including the REST corepressor (Rcor)-2 factor, which seems to be involved in an inflammatory suppressive program.

**Methods:**

To assess the impact of *Rcor2* in age-related inflammation, gene expression levels were quantified in different tissues and ages of the spontaneous senescence-accelerated P8 mouse (P8) using the SAMR1 mouse (R1) as a control. Specific siRNA transfection in P8 and R1 astrocyte cultures was used to determine *Rcor2* involvement in the modulation of neuroinflammation. The effect of lipopolysaccharide (LPS) treatment on *Rcor2 levels* and neuroinflammation was analyzed both *in vivo* and *in vitro*.

**Results:**

P8 mice presented a dramatic decrease in *Rcor2* gene expression compared with R1 controls in splenocytes, an alteration also observed in the brain cortex, hippocampus and primary astrocytes of these mice. *Rcor2* reduction in astrocytes was accompanied by an increased basal expression of the interleukin (*Il*)-6 gene. Strikingly, intraperitoneal LPS injection in R1 mice downregulated *Rcor2* in the hippocampus, with a concomitant upregulation of tumor necrosis factor (*Tnf-α*), *Il1-β* and *Il6* genes. A negative correlation between *Rcor2* and *Il6* gene expression was also verified in LPS-treated C6 glioma cells. Knock down of *Rcor2* by siRNA transfection (siRcor2) in R1 astrocytes upregulated *Il6* gene expression while siRcor2 further increased *Il6* expression in P8 astrocytes. Moreover, LPS activation provoked a further downregulation *of Rcor2* and an amplified induction of Il6 in siRcor2-tranfected astrocytes.

**Conclusions:**

Data presented here show interplay between *Rcor2* downregulation and increased inflammation and suggest that *Rcor2* may be a key regulator of inflammaging.

## Background

The process of aging is associated with a low-grade systemic inflammation defined as inflammaging [1], which is characterized by an increase of acute-phase circulating proteins and proinflammatory factors such as interleukin (IL)-6 and tumor necrosis factor (TNF-α). This condition has been connected with the onset and evolution of age-related diseases, including type 2 diabetes mellitus, cardiovascular disease, cancer and neurodegeneration [2-4]. Indeed, the aging brain exhibits a progressive increase in inflammatory processes [5], which can be neurotoxic, altering important central functions [6] and contributing to the pathogenesis of neurodegenerative disorders such as Alzheimer’s disease (AD) [7]. The microglia plays a critical role in neuroinflammation as it represents the main type of immune cells in the brain [8]. The activation of microglia in aging models leads to the secretion of proinflammatory cytokines, which contribute to synaptic and neuronal damage as well as cognitive impairment [9,10].

Recent studies using diverse experimental models have described the role of chromatin regulators in inflammation. Among them, REST corepressor (RCOR) factor seems to be involved in an inflammatory suppressive program. In mammals, two *Rcor* gene isoforms (*Rcor1/CoREST and Rcor2*) have so far been studied. RCOR is found in a complex with the histone demethylase lysine-specific demethylase 1A (KDM1A, previously known as LSD1), which exhibits nucleosome demethylase activity [11-13], and with the RE1-silencing transcription factor (REST), which has been recently associated with cognitive preservation and longevity [14]. RCOR1/CoREST is involved in the modulation the expression of neuronal genes [15] while RCOR2 plays a role in embryonic stem cells (ESCs) maintenance and regulation [13]. We have recently shown that knock down of LSD1/KDM1A or its partner, RCOR2, in pre-adipocytes results in derepression of an inflammatory program with a significant increase in the expression of inflammatory genes such as *Il6*, chemokine ligand 2 (*Ccl2*), macrophage proinflammatory protein 1α (*Mip1a*, also known as *Ccl3*), chemokine (C-X-C motif) ligands 5 and 10 (*Cxcl5* and *Cxcl10*), and macrophage activation 2-like (*Mpa2l*) [16]. Consistent with these findings, RCOR was also found to mediate the turnover of nuclear factor kappa B (NF-κB) and to restore LPS-activated genes to a basal expression in microglia and astrocytes [17].

Here, we examined the link between *Rcor2* and inflammation in the SAMP8 mouse model of accelerated aging and neurodegeneration. The SAMP8 strain (P8) was selected from AKR/J mice and is a well-characterized model for studying pathological brain aging [18-21]. The SAMR1 mice (R1), with a similar genetic background and normal aging characteristics, represent a suitable and widely used control model [22]. P8 mice presented signs of accelerated aging such as loss of activity, skin coarseness, alopecia, lack of hair glossiness, increased lordokyphosis, periophthalmic lesions, and systemic senile amyloidosis [23]. These mice also displayed cognitive and behavioral alterations that were accompanied by molecular features typical of Alzheimer’s disease (AD), such as overproduction of amyloid-beta protein, increased tau phosphorylation, cholinergic deficits in the forebrain and increased oxidative stress [18,19,24-28]. Biomarkers of inflammation such as C-reactive protein and serum amyloid P are elevated in P8 mice [29].

Here we found that P8 mice present a spontaneous decrease in *Rcor2* gene expression, which is, at least in part, responsible for the inflamed phenotype in astrocytes. Our data show that *Rcor2* downregulation and neuroinflammation are mutually influenced processes.

## Materials and methods

### Animal care

All experimental procedures were approved by the Ethics Committee of the Autonomous University of Barcelona (Comissió Ètica d’Experimentació Animal i Humana, CEEAH, UAB), following the ‘Principles of laboratory animal care’, and were performed in accordance with the European Communities Council Directive (86/609/EEC). SAMP8 and SAMR1 female mice were provided by the Parc Cientific, (University of Barcelona, Spain) and were maintained under standard conditions (temperature 23 ± 1°C, humidity 50 to 60%, 12:12-h light-dark cycle, lights on at 7:00 a.m.), with food (A04, Harlan, Spain) and tap water available *ad libitum* throughout the study. Body weight (g) was measured weekly. For tissue harvesting, mice were sacrificed by cervical dislocation. The hippocampi and cerebral cortices were immediately frozen and stored at -80°C for further analysis. The spleen was extracted and sliced, and splenocytes were isolated through a 70-μm cell strainer (BD Biosciences, San Jose, CA) in PBS 1X and collected following centrifugation at 1600 rpm for 5 minutes. Erythrocytes were further lysed using BD Pharm Lyse™ solution (BD Biosciences, San Jose, CA) at 37°C for 2 min. Cells were recovered by centrifugation and pellets were aliquoted and frozen at -80°C for further analysis.

### Lipopolysaccharide treatment and cytokine determination

SAMP8 and SAMR1 mice (males, 12-months old, n = 3/group) received an intraperitoneal (IP) injection of lipopolysaccharide (LPS) (2.9 mg/kg; Sigma-Aldrich, St. Louis, Mo., USA), or an equal volume of saline. Three hours after treatment, blood samples were collected and mice were sacrificed by cervical dislocation. Tissues were collected and immediately frozen in liquid nitrogen and stored at -80°C until processed.

Detection of TNF-α, IL1-β and IL6 in plasma was performed by enzyme-linked immunosorbent assay, in accordance with the protocol provided by the manufacturer (eBioscience, San Diego, CA; R&D Systems, Minneapolis, MN and Diaclone, Besançon, France respectively).

### Cell culture and transfection

Primary cultures of astrocytes were established from cerebral cortical tissue of 2-day-old SAMR1 and SAMP8, as previously described [30]. Cortices were promptly dissected free of the meninges, diced into small cubes, and dissociated by incubation for 25 min with a 0.25% trypsin/1 mM EDTA solution (Gibco-Invitrogen, Carlsbad, CA). After a further mechanical dissociation, cells were resuspended in Dulbecco’s Modified Eagle’s Medium supplemented with 2.5 mM glutamine, 100 g/mL gentamycin, and 20% FBS (Gibco-Invitrogen, Carlsbad, CA). Cells were seeded at 5×10^4^ cell/cm^2^ in T75 flasks and incubated at 37°C in a humidified atmosphere of 5% CO_2_ - 95% air. The culture medium was changed twice a week. The concentration of FBS was changed to 15% and 10% after 1 and 2 weeks of culture, respectively. After 3 weeks, the flasks were shaken in an orbital shaker at 200 rpm for 4 h to dislodge microglia and, subsequently, the attached astrocyte monolayer was collected. This purified fraction contained more than 90 to 95% of astrocytes, with a minor fraction of contaminating microglia.

Astrocytes were trypsinized 1 h before transfection, distributed into 48-well plates (1 × 10^5^ cells/well) and subsequently transfected with 60 nM of double-stranded siRNAs using Metafectene Pro (Biontex, Martinsried, Germany) at a 1:1 ratio with the siRNA. The sequences of the siRNAs used were: 5’-GGUCUUGACUCACAGCUCAtt-3’ (Sense strand) and 5’-UGAGCUGUGAGUCAAGACCtc-3’ (Antisense strand) for siRCOR2; and Silencer™ negative control siRNA #1 (Applied Biosystems, Foster City, CA) for siC. RNA was extracted 48 h after transfection.

Rat C6 glioma cells (purchased from the European Collection of Cell Cultures, ECACC) were cultured as monolayer and grown in F12/DMEM medium (Gibco-Invitrogen, Carlsbad, CA) supplemented with 10% fetal bovine serum (Gibco-Invitrogen, Carlsbad, CA), 1% non essential a/a’s (Gibco-Invitrogen, Carlsbad, CA), 1/500 gentamicin (Gibco-Invitrogen, Carlsbad, CA) and maintained at 37°C and 5% CO_2_ in a humidified environment.

For each treatment, C6 cells were seeded in a six-well plate and cultured in F12/DMEM without serum for 12 h prior to stimulation with LPS. Cells were incubated with LPS (1 μg/ml) for different time periods ranging from 30 min to 6 h.

### Total RNA extraction

Total RNA was extracted from frozen tissues through mirVana™ RNA Isolation Kit (Applied Biosystems, Foster City, CA) in accordance with the manufacturer’s instructions.

RNA extraction from astrocytes and C6 cultured cells was performed using TRIzol™ Reagent (Invitrogen, Carlsbad, CA) in accordance with the manufacturer’s protocol.

The yield, purity, and quality of RNA were determined spectrophotometrically (Nano-drop Technologies, Wilmington, DE) and using the Bioanalyzer 2100 capillary electrophoresis (Agilent Technologies, Palo Alto, CA).

RNA samples with a 260/280 ratio and an RIN higher than 1.9 and 7.5, respectively, were selected.

### Real-time quantitative polymerase chain reaction

Random-primed cDNA synthesis was performed at 37°C starting with 0.3 μg of RNA, using the High Capacity cDNA Archive kit (Applied Biosystems, Foster City, CA). Gene expression was measured in an ABI Prism 7900HT Real Time PCR system using TaqMan FAM labeled specific probes (Applied Biosystems, , Foster City, CA [see Additional file 1]). Results were normalized to the housekeeping TATA-binding protein (*Tbp*) gene.

### Statistical analysis

The statistical analysis was carried out using the Statistical Package for Social Sciences (SPSS, version 19.0, http://www.ibm.com/software/es/analytics/spss/). An analysis of variance (ANOVA) was conducted to assess the effects of strain and specific conditions. Comparisons between groups were performed by the two-tailed Student’s t-test for independent samples or the Mann-Whitney U-test as indicated.

Partial correlation (controlling for time of exposure to LPS) was used to examine the relationship between *Il-6* and *Rcor2* expression in the C6 glial cell line.

All values were expressed as mean ± standard error of the mean. *P* <0.05 was considered significant. Statistical outliers (≥two standard deviations from the mean) were removed from analyses.

## Results and discussion

### Increased levels of IL6 in serum of P8 mice after intraperitoneal lipopolysaccharide administration

**Figure 1:**
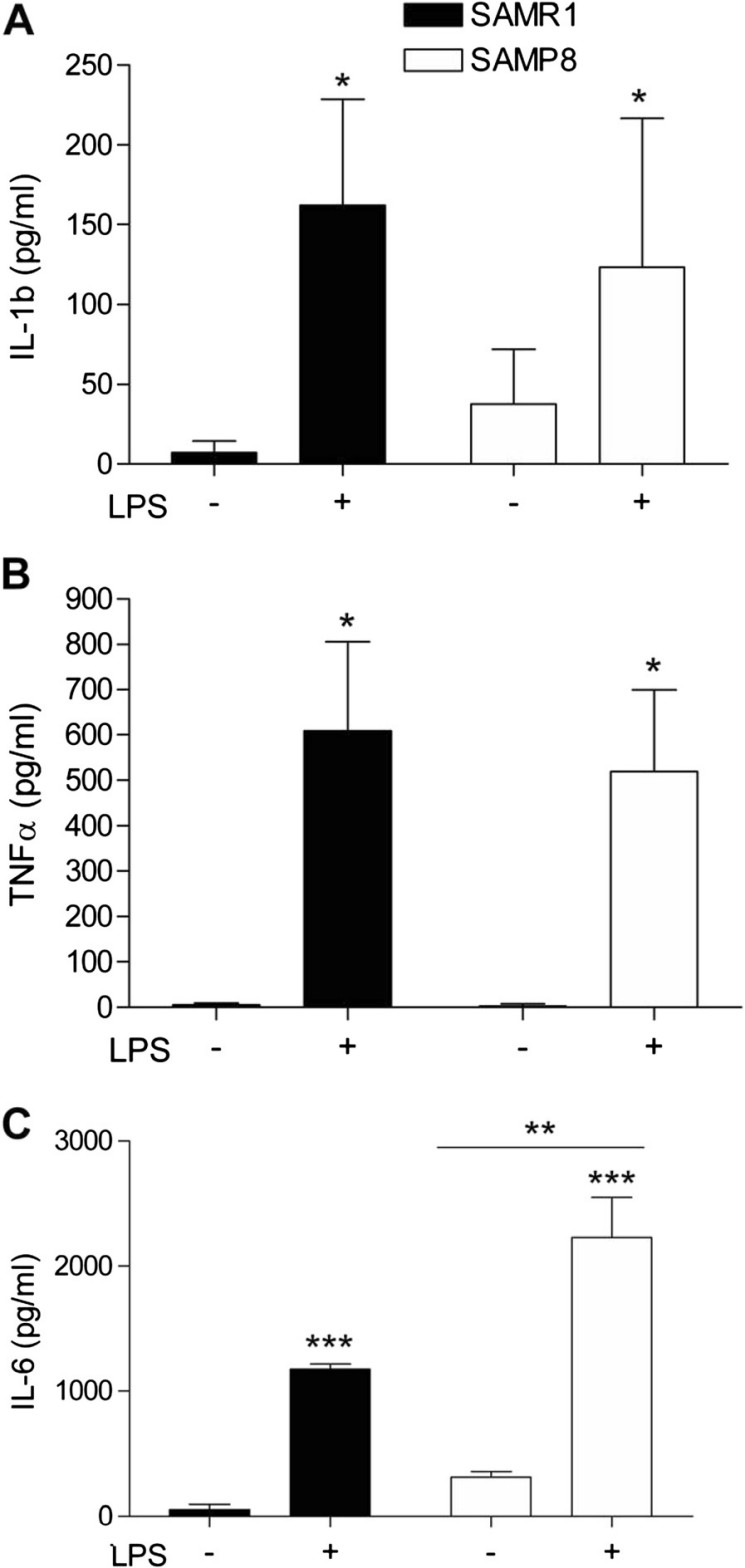
**Inflammatory response in serum from R1 and P8 mice after lipopolysaccharide (LPS) administration. (A)** Interleukin 1 beta, **(B)** TNF-α serum levels, **(C)** Interleukin 6. Cytokine were measured by ELISA (n = 3/group). Mean ± standard error are represented; Two-way ANOVA results are indicated as *P <0.05; **P <0.01; ***P <0.001.

P8 senescent mice exhibited signs of systemic inflammation affecting the liver, pancreas, cochlea, bones, testes and plasma levels of several cytokines and proinflammatory factors [29,31-37]. In addition to their peripheral inflamed phenotype, a proinflammatory brain environment may be involved in the neurodegenerative traits and/or cognitive deficiency in P8 mice [38]. The inflammatory response was analyzed three hours after LPS intraperitoneal injection in 12 month-old P8 mice and age-matched R1 controls. A pronounced increase was found in IL1β, TNFα and IL6 serum levels both in P8 and R1 mice in response to LPS (IL1 β: F(1,7) = 0.962, *P* <0.05; TNFα: H(3) = 8.389, *P* <0.05; IL6: F(1,7) = 67.362, *P* <0.001) (Figure 1A and B). Neither strain effect nor interaction between strain and LPS treatment was observed for IL1β and TNFα cytokines. In contrast, we found both strain and LPS effects for IL6 (F(1,7) = 12.706, *P* <0.01; F(1,7) = 67.362, *P* <0.001) as well as a strong tendency for interaction between strain and treatment (*P* = 0.068). Moreover, IL6 showed increased basal levels in P8 mice (t(3) = -4.139; *P* <0.05) (Figure 1C). These data suggest that plasma IL6 could be an inflammatory biomarker of P8 inflamed phenotype.

### Decreased *Rcor2* expression in P8 splenocytes, cortex, hippocampus and astrocytes

**Figure 2:**
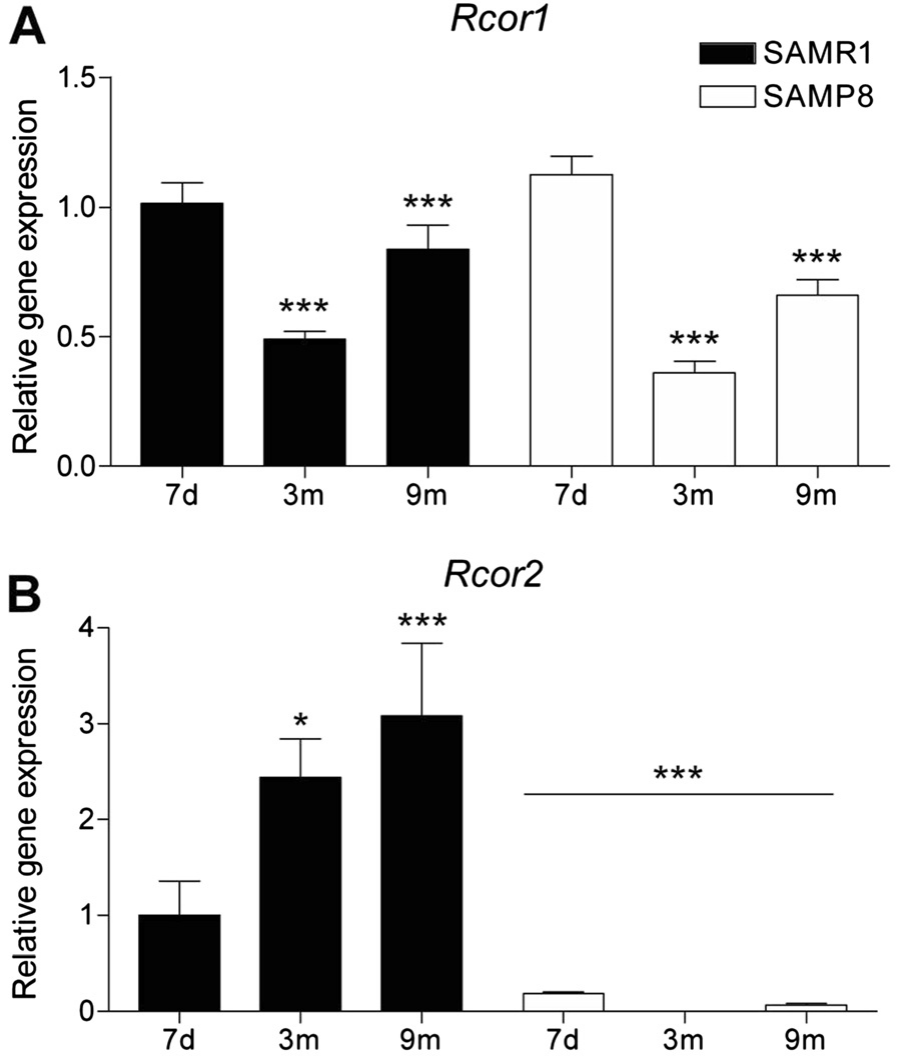
***Rcor*gene expression profile at different ages in splenocytes from R1 and P8 mice. (A)***Rcor1* and **(B)***Rcor2* gene expression at the ages of 7 days, 3 months and 9 months (n = 3 to 10/group). Gene expression levels were determined by real time PCR using TaqMan FAM-labeled specific probes and expressed relative to *Tbp*. Mean ± standard error are represented; Two-way ANOVA results are indicated as *P <0.05; ***P <0.001.

We explored the expression of *Rcor1* and *Rcor2* in P8 mice with the aim of elucidating a possible mechanism for their inflamed phenotype and increased response to LPS treatment. We analyzed the *Rcor* gene expression pattern in splenocytes from P8 and R1 mice at three different ages: 7 days, 3 months and 9 months. Both strains presented similar age-dependent gene expression patterns for *Rcor1* with higher levels at 7 days and 9 months compared with 3 months (F(2,22) = 32.306, *P* <0.001) (Figure 2A). In contrast, R1 mice showed an age-dependent increase in *Rcor2* gene expression which was not observed in P8 mice (interaction between strain and age: F(2,29) = 4.279, *P* <0.05) (Figure 2B). Intriguingly, P8 mice exhibited low expression levels of *Rcor2* from the first week of age and throughout the adult life. These data indicate that *Rcor2* underexpression in splenocytes precedes most of the accelerated-aging characteristics of this model. Indeed, we found a marked effect for strain and age in *Rcor2* gene expression (F(1,29) = 31.620, *P* <0.001; F(2,29) = 3.363, *P* <0.001; respectively).

**Figure 3:**
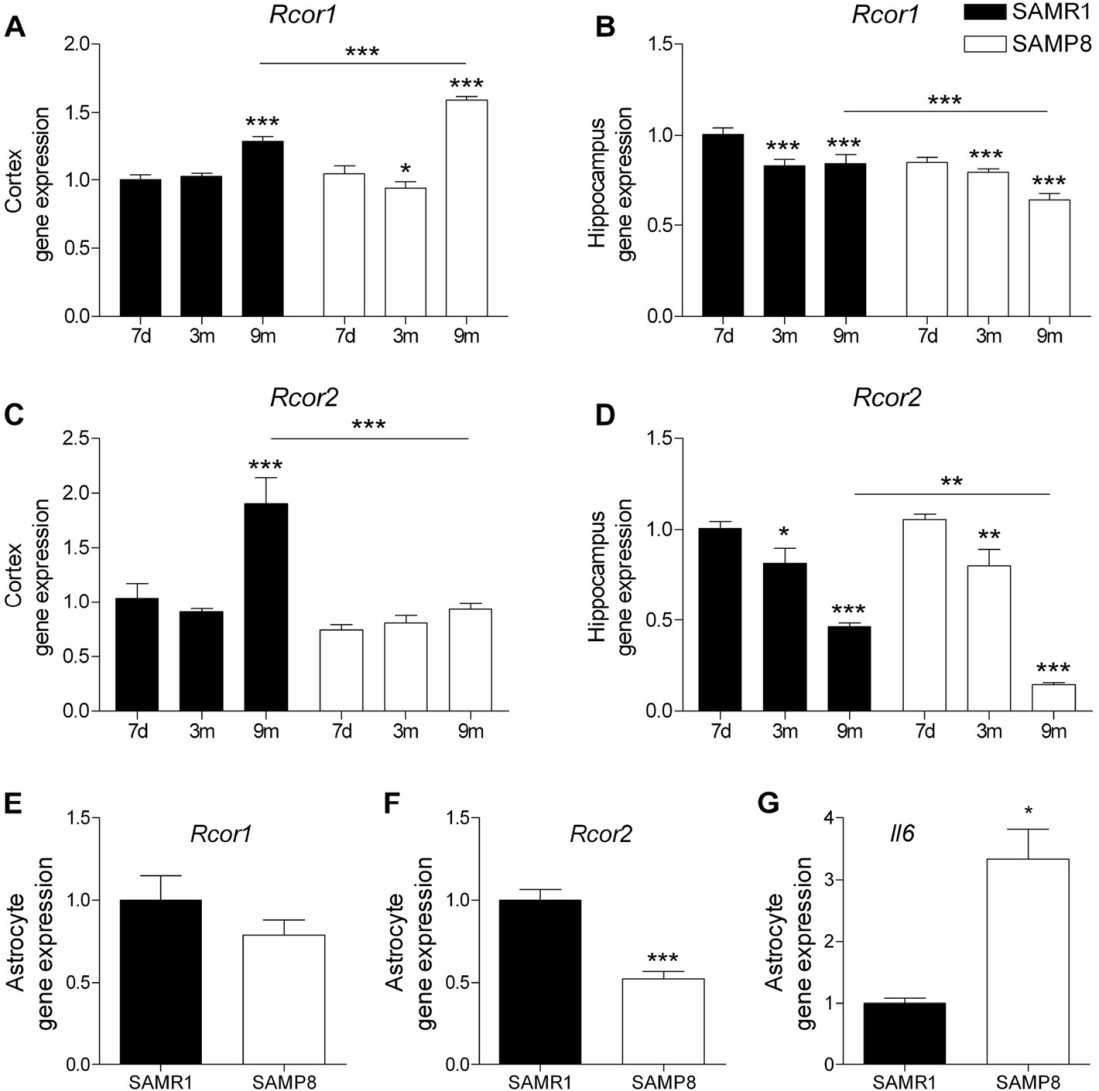
***Rcor*gene expression profile in brain tissues from R1 and P8 mice.***Rcor1* and *Rcor2* gene expression was quantified in the cortex **(A and C)**, in the hippocampus **(B and D)** at the ages 7 days, 3 months and 9 months (n = 4 to 8/group), and in primary cultured astrocytes (n = 6 to 9/group) **(E and F)** from 2 days-old mice. **(G)** Interleukin 6 basal gene expression levels in primary cultured astrocytes obtained from 2-day-old mice (n = 4/group). Gene expression levels were measured by real time PCR. Mean ± standard error are represented; Two-way ANOVA results are indicated as *P <0.05; **P <0.01; ***P <0.001.

Chronic inflammation is associated with early development and acceleration of neurodegenerative diseases such as Alzheimer’s disease [39,40]. Therefore, we analyzed the expression of *Rcor1* and *Rcor2* in P8 cortices and hippocampi. *Rcor1* presented age effect in both cortex and hippocampus tissues (F(2,27) = 49.140, *P* <0.001; F(2,32) = 12.597, *P* <0.001; respectively) with a similar age-dependent expression pattern in both strains (Figure 3A and B). However, 9-month-old P8 mice, unlike R1 mice, presented slight but significant alterations in *Rcor1* levels in the cortex and in the hippocampus. On the other hand, *Rcor2* was expressed similarly in R1 and P8 brain cortices and hippocampi at 7 days and 3 months of age (Figure 3C and D).

Remarkably, a consistent characteristic among splenocytes, cortex and hippocampus was that *Rcor2* gene expression in 9-month-old P8 mice decreased significantly in comparison with R1 controls (cortex: *P* <0.001; hippocampus: *P* <0.01).

We also explored the gene expression of *Rcor1* and *Rcor2* in P8 and R1 astrocytes obtained from cerebral cortical tissue of 2-day-old mice. P8 cells showed similar *Rcor1* but decreased *Rcor2* gene expression in comparison with R1 cells (t(11) = 5,837; *P* <0.001) (Figure 3E and F). Notably, the reduced levels of *Rcor2* in P8 compared with R1 astrocytes were accompanied by increased basal levels of *Il6* gene expression (t(3) = -4.734; *P* = 0.016) (Figure 3G).

These data show that *Rcor2* is underexpressed in different tissues and organs in P8 mice and, in some cases (namely, in splenocytes and astrocytes), this alteration is detected from early ages. Interestingly, Yankner and colleagues have recently shown that the transcriptional repressor REST (RE1-silencing transcription factor) is crucial for neuroprotection during aging [14]. It is known that REST recruitment of RCOR corepressor is required for its gene silencing activity [15]. The downregulation of *Rcor2* in immune and brain cells of the P8 senescent mice strongly suggests that the accelerated aging in these mice, as well as their neurodegenerative phenotype, may be related to *Rcor2* deficiency.

On the other hand, RCOR is part of a chromatin remodeling complex containing the lysine specific demethylase (KDM1A), which catalyzes H3 histone demethylation as a way to repress transcription [12]. In the absence of RCOR, KDM1A demethylase activity is impaired with a concomitant increase in H3 histone methylation levels. In this context, preliminary data from our group indicate that the alteration in histone methylation levels is a candidate mechanism through which *Rcor2* could also influence the aging process in P8 mice. Indeed, P8 mice present increased levels of trimethylated histone H3 lysine 4 (H3K4me3) in splenocytes, cortex, and hippocampus (supplementary information, [see Additional file 2A-C]). Moreover, consistent with a role of *Rcor* in methylation signaling and in neuroinflammation, we have found that *Il6* upregulation in response to LPS is significantly dependent on methyltransferase activity (supplementary information, [see Additional file 3]).

### Negative correlation between R*cor2* and inflammatory gene expression after lipopolysaccharide (LPS) treatment in C6 glioma cells and mouse hippocampus

**Figure 4:**
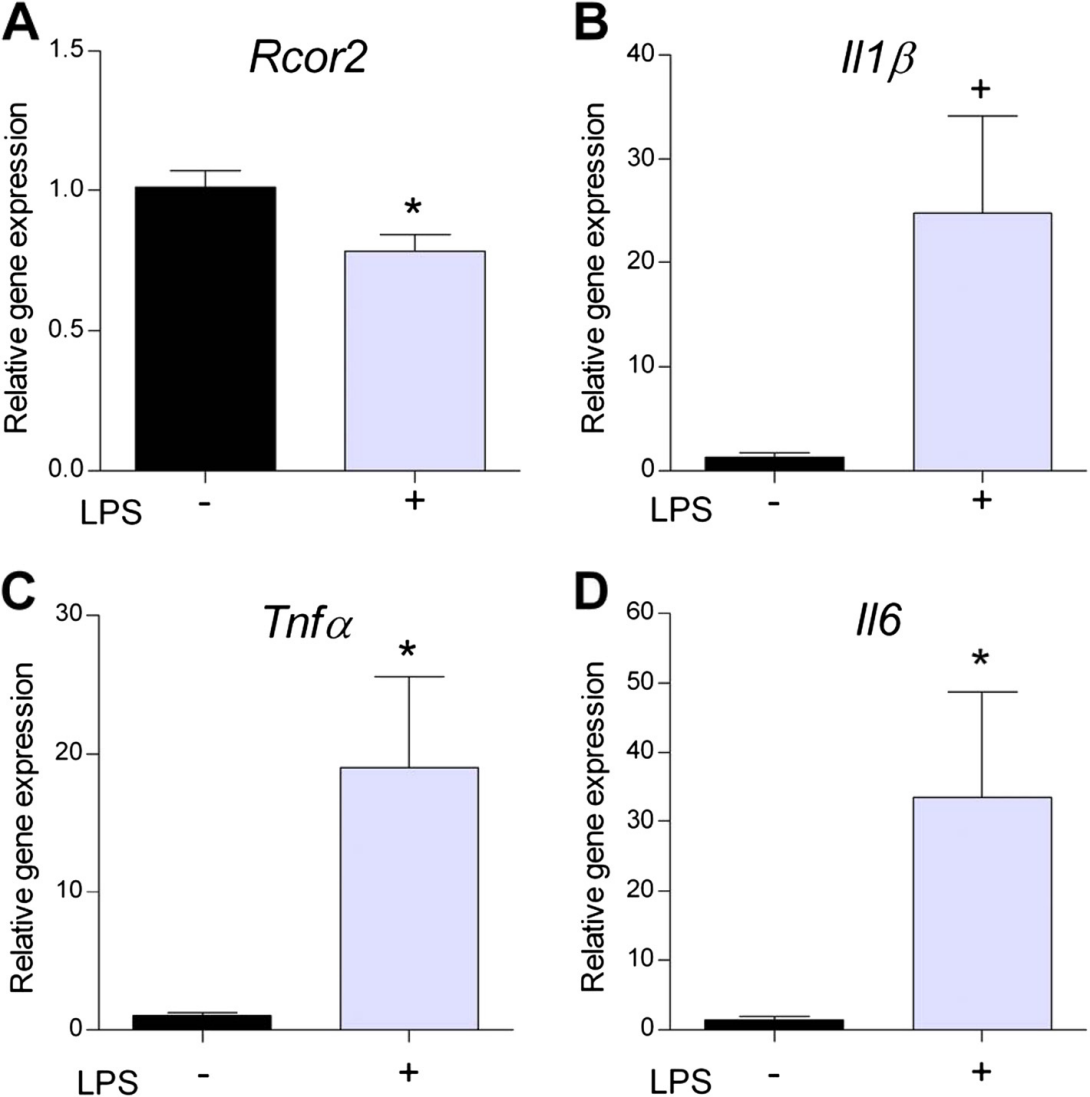
**Concomitant downregulation of *Rcor2 *and increased pro-inflammatory gene expression in the R1 hippocampus after intraperitoneal lipopolysaccharide (LPS) injection. (A)***Rcor2***(B)** interleukin 1 beta, **(C)** tnf-alpha and) **(D)** interleukin 6 gene expression levels in hippocampus from 12 month-old R1 after intraperitoneal lipopolysaccharide (LPS) injection (n = 5 to 7/group). Real-time PCR was performed and means ± standard errors are represented. Two-way ANOVA results are indicated as *P <0.05.

To approach the possible role of *Rcor2* in neuroinflammation, we first analyzed its gene expression after an *in vivo* intraperitoneal LPS injection in 12-month-old P8 and R1 mice. While P8 hippocampus was not further responsive to the peripheral LPS treatment [see Additional file 4], R1 mice presented a significant decrease in *Rcor2* expression (t(12) = 2.606, *P* = 0.023) together with an increase in *Tnfα*, *Il1*β and *Il6* gene expression levels (t(5.01) = -2.726, *P* <0.05; t(5.02) = -2.490, *P* = 0.055; U(9) = 0.000, Z = -2.449, *P* <0.05; respectively) (Figure 4A-D).

**Figure 5:**
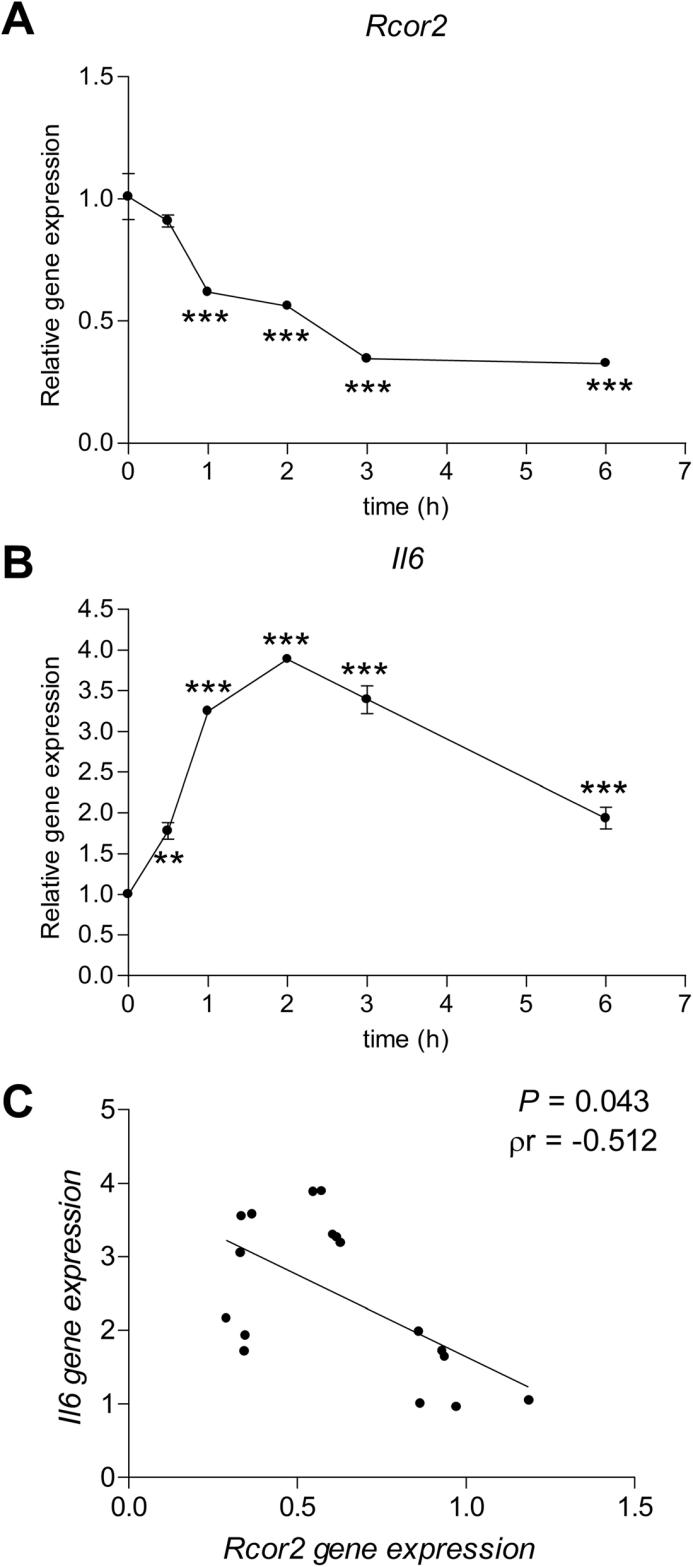
**Concomitant downregulation of *Rcor2 *and increased *IL6 *gene expression in lipopolysaccharide (LPS)-treated C6 glial cell line. (A)***Rcor2* and **(B)** interleukin 6 gene expression after LPS stimulation (1 μg/ml) in C6 glial cells. Gene expression levels were determined by real-time PCR. Mean ± standard error from three independent experiments are represented. One-way ANOVA results are indicated as **P <0.01; ***P <0.001. **(C)** Partial correlation between *Rcor2* and *Il6* gene response after LPS treatment in C6 glial cells. P value (*P*) and partial correlation coefficient (ρr) are indicated.

A similar effect was found in C6 glioma cells treated with LPS. C6 cells were activated with LPS for 30 min and 1, 2, 3 and 6 hours. We found a significant reduction in *Rcor2* gene expression (F(5,12) = 47.332; *P* <0.001) with a concomitant increase in *Il6* expression in response to LPS (F(5,11) = 106.833; *P* <0.001) (Figure 5A and B, respectively). A significant correlation (controlling for time of exposure to LPS) was found between *IL-6* and *Rcor2* expression (ρr = -0.512 and *P* = 0.043) (Figure 5C).

### Knock down of *Rcor2* in astrocytes upregulates *Il6* gene expression

To determine the involvement of *Rcor2* in the modulation of *Il6* expression, we knocked down *Rcor2* in R1 and P8 astrocytes by transfection of a siRNA to *Rcor2* (siRcor2) using a scrambled siRNA as a control (siC).

**Figure 6:**
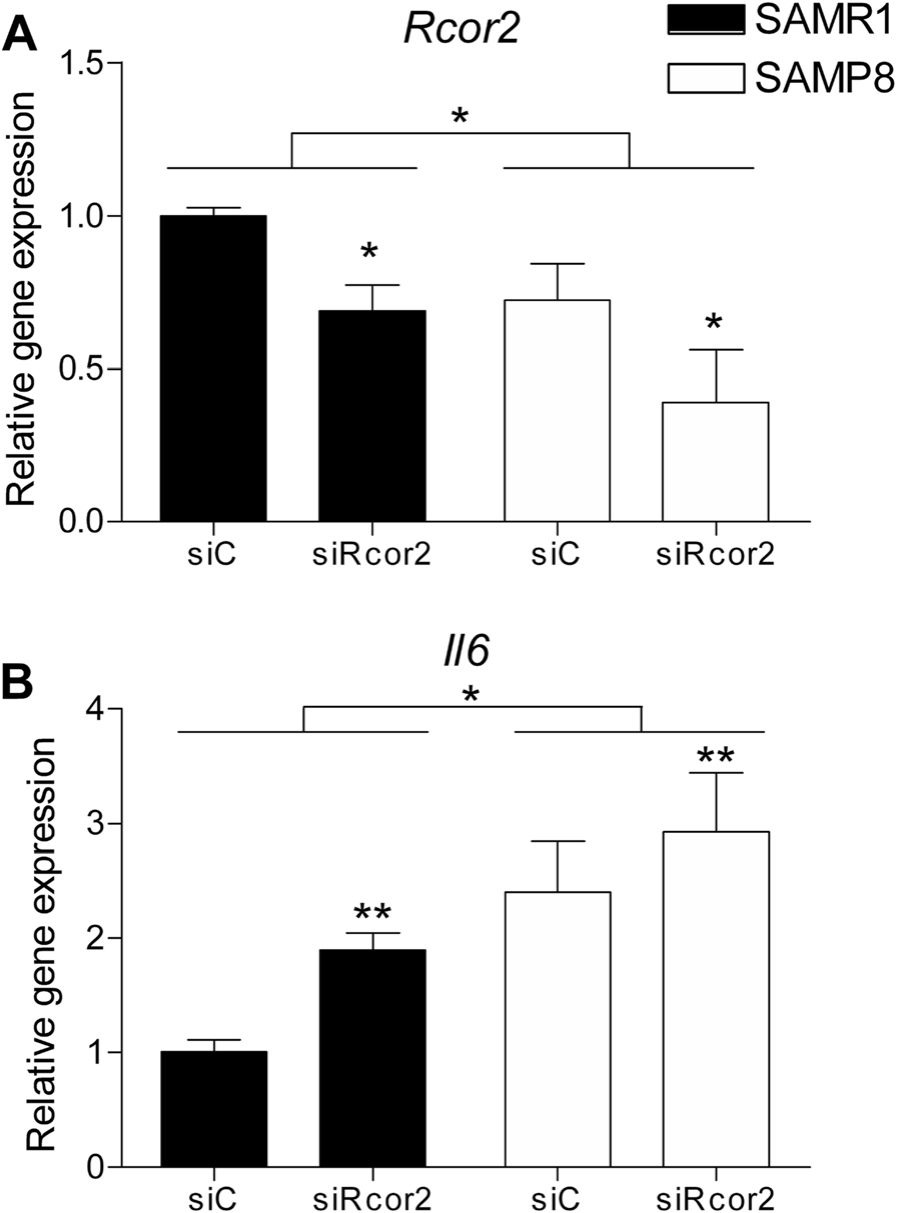
**Knock down of *Rcor2 *in P8 and R1 astrocytes. (A)***Rcor2* and **(B)** Interleukin 6 basal gene expression levels after transfection in P8 and R1 astrocytes of a siRNA to *Rcor2* (siRcor2) using a scrambled siRNA as a control (siC). Gene expression levels were measured by real-time PCR. Mean ± standard error from three independent experiments are represented; Two-way ANOVA results are indicated as *P <0.05.

*Rcor2* knock down led to a 31% and 46% decrease in its expression levels in R1 and P8 astrocytes, respectively (F(1,7) = 10.082, *P* = 0.016) (Figure 6A). Moreover, si*Rcor2* astrocytes showed increased *Il6* levels in both strains with significantly higher *Il6* levels in P8 compared with the R1 cells (strain: F(1,6) = 10.022, *P* <0.05) (Figure 6B).

Knock-down data in R1 and P8 astrocytes reinforce the notion that *Rcor* gene product acts as a repressor of an inflammatory program [16,17]. Our findings suggest that *Rcor2* underexpression may be at the root of the inflamed phenotype that characterizes the P8 strain [29].

### Interplay between *Rcor2* expression and inflammatory response in si*Rcor2* astrocytes

*Il6* gene expression was then analyzed in R1 and P8 astrocytes transfected with si*Rcor2* following activation by LPS and Interferon gamma (IFNγ). Astrocytes from both strains transfected with scrambled siRNA (siC) were used as controls.

**Figure 7:**
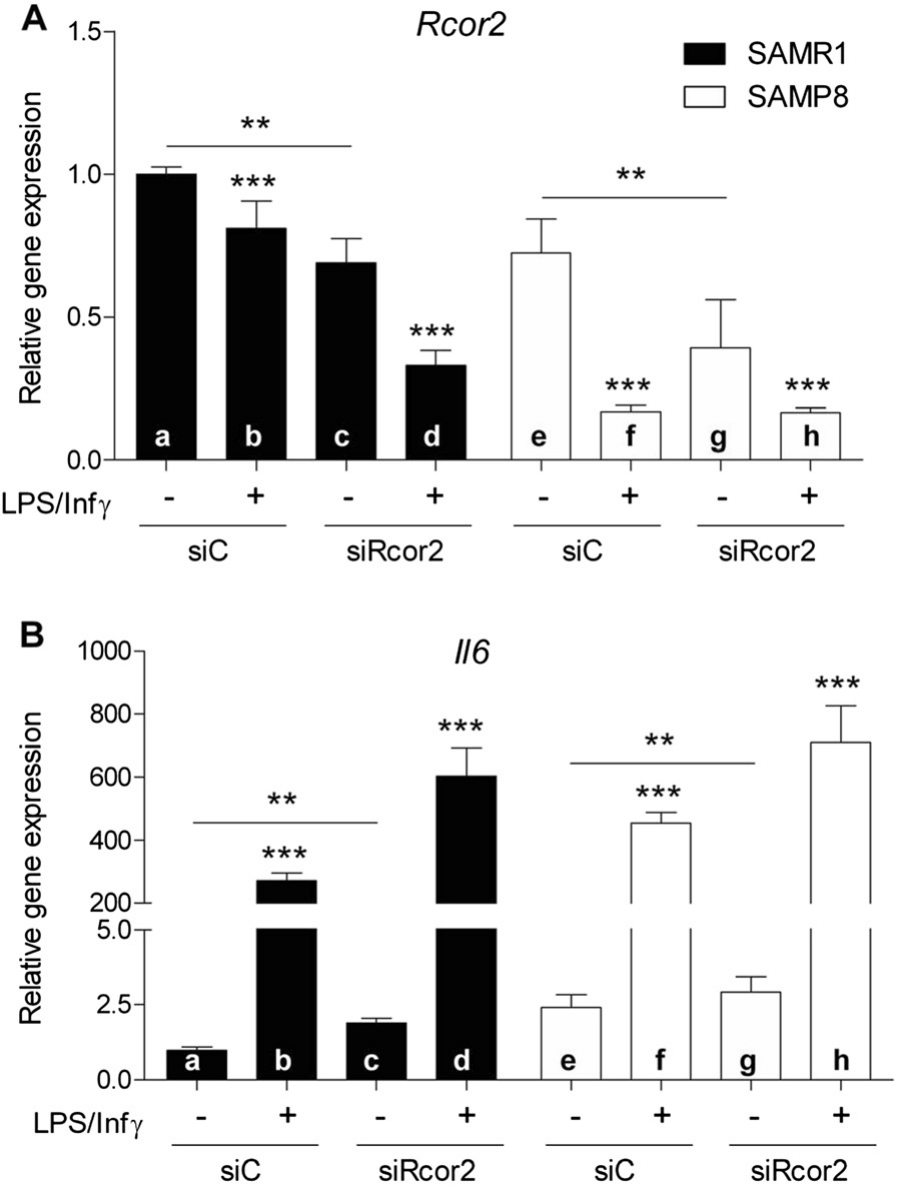
**Interplay between *Rcor2 *expression and inflammatory response in si *Rcor2 *astrocytes. (A) ***Rcor2* and **(B)** interleukin 6 gene expression levels after 4.5 h lipopolysaccharide (LPS) (2 μg/ml) and interferon-gamma (3 μg/ml) stimulation in R1 and P8 astrocytes transfected with siRNA to *Rcor2* (siRcor2) or scrambled siRNA as a control (siC) (n = 3/group). Gene expression levels were determined by real-time PCR. Mean ± standard error are represented; Two-way ANOVA results are indicated as **P <0.01; ***P <0.001.

Notably, LPS/IFNγ treatment further reduced *Rcor2* expression (F(1,14) = 47.465, *P* <0.001) with a concomitant increase in *Il6* expression in all conditions tested (siC and siRcos2 transfected R1 and P8 cells) (F(1,14) = 134.730, *P* <0.001) (Figure 7B) (Figure 7A and B, a versus b; c versus d; f versus e; and g versus h).

For *Rcor2*, the interaction between strain and LPS/IFNγ stimulation was also significant (F(1,14) = 16.673, *P <*0.01) indicating that the treatment led to a greater decrease in *Rcor2* after LPS/IFNγ stimulation in P8 than in R1 cells. *Il6* expression in response to the stimulus was significantly exacerbated in siRcor2 knock down astrocytes in comparison with siC-transfected cells (transfection X treatment effect: F(1,14) = 11.311, *P* <0.01) (Figure 7B, d *versus* b and h *versus* f).

These results suggest that *Rcor2* downregulation in astrocytes induces inflammation and in turn, an inflammatory environment further downregulates *Rcor2*. We can conclude from our data on transfected astrocytes that the LPS/INFγ stimulus and *Rcor2* knock down represent independent modulators of Il6 expression and show a synergic effect when combined.

## Conclusions

Little information is currently available on the molecular triggers of chronic low-grade inflammation, a process that affects the function of most organs and tissues including the central nervous system and is involved in aging and neurodegeneration [41,42].

RCOR is found in a complex with RE1-silencing transcription factor (REST), which plays a role in protecting the brain from age-related insults in animal models and a role in preservation of cognitive function and longevity in aging humans [14]. Taking into account the early downregulation of RCOR2 in the P8 mouse model of accelerated aging and neurodegeneration, further studies are warranted so as to examine whether the REST/RCOR complex is involved in the shorter lifespan of P8 mice as well as the role of RCOR2 in human cognition and longevity.

Our study indicates that neuroinflammation is a pathophysiological outcome of *Rcor2* downregulation in the SAMP8 mice. The fact that *Rcor2* and *Il6* presented an opposite response after an inflammatory stimulus in hippocampus and in glial cells, suggests a possible interplay between the pathways involved in the regulation of both genes. This notion is reinforced by our data showing that *Il6* gene is upregulated by *Rcor2* knock down in astrocytes. Therefore, RCOR2 may represent a potential target for the prevention or treatment of age-related chronic inflammation and neurodegeneration.

## Abbreviations

Rcor: REST corepressor; P8: senescence-accelerated prone 8 mouse; R1: senescence-accelerated resistant 1; LPS: lipopolysaccharide; IL: interleukin; *Tnf-α*: tumor necrosis factor; AD: Alzheimer’s disease; KDM1A: histone demethylase lysine-specific demethylase 1A; REST: RE1-silencing transcription factor; ESCs: embryonic stem cells; *Ccl2*: chemokine ligand 2; *Mip1a*: macrophage proinflammatory protein 1α; *Cxcl*: chemokine (C-X-C motif) ligands; NF-κB: nuclear factor kappa B; IFNγ: Interferon gamma; Rcor: REST corepressor; P8: senescence-accelerated prone 8 mouse; R1: senescence-accelerated resistant 1; LPS: lipopolysaccharide; IL: interleukin; *Tnf-α*: tumor necrosis factor; AD: Alzheimer’s disease; KDM1A: histone demethylase lysine-specific demethylase 1A; REST: RE1-silencing transcription factor; ESCs: embryonic stem cells; *Ccl2*: chemokine ligand 2; *Mip1a*: macrophage proinflammatory protein 1α; *Cxcl*: chemokine (C-X-C motif) ligands; NF-κB: nuclear factor kappa B; IFNγ: Interferon gamma.

## Competing interests

The authors declare that they have no competing interests.

## Authors’ contributions

Designed research: MJAL and PK; performed research: MJAL, PMM, MCF, MCT, MPar, RC, RME, MP and PK; contributed new reagents/analytic tools: MPar, RME, MP, CS and PK; analyzed data MJAL, MCF, MCT, MPar, RC, CS, and PK; wrote the manuscript: MJAL and PK. All authors read and approved the final manuscript.

## Supplementary Material

Additional file 1 List of primers and probe sets used for real time RT-PCR analysis. Click here for file

Additional file 2 Histone H3 methylation pattern in R1 and P8 tissues. Click here for file

Additional file 3 Methyltranferase inhibitor MTA reduces lipopolysaccharide (LPS)-induced Il6 gene expression in C6 glioma cells. Click here for file

Additional file 4 Pro‒inflammatory gene expression response in P8 hippocampus after intraperitoneal lipopolysaccharide (LPS) injection. Click here for file
