# Supplementary material for: Rcor2 underexpression in senescent mice: a target for inflammaging?
Source: J Neuroinflammation. 2014 Jul 23;11:126. doi: 10.1186/1742-2094-11-126 (PMC4128581; doi:10.1186/1742-2094-11-126)
Supplement: Additional file 1 — List of primers and probe sets used for real time RT-PCR analysis. [file 1742-2094-11-126-S1.docx]

| Gene Symbol | Specie | Reference (ABI) | Reference Sequence ID |
| --- | --- | --- | --- |
| *Il1β* | Mouse | Mm00434228_m1 | NM_008361.3 |
| *Il6* | Mouse | Mm00446191_m1 | NM_031168.1 |
| *Rcor1* | Mouse | Mm01336704_m1 | NM_198023.2 |
| *Rcor2* | Mouse | Mm00499756_m1 | NM_054048.3 |
| *Tnfα* | Mouse | Mm00443258_m1 | NM_013693.2 |
| *Tbp* | Mouse | Mm00446971_m1 | NM_013684.3 |
| *Il6* | Rat | Rn01410330_m1 | NM_012589.1 |
| *Rcor2* | Rat | Rn01454655_g1 | NM_001013994.1 |
| *Tbp* | Rat | Rn01455646_m1 | NM_001004198.1 |

Additional file 1. List of primers and probe sets used for real time RT-PCR analysis.
