# Supplementary material for: Rcor2 underexpression in senescent mice: a target for inflammaging?
Source: J Neuroinflammation. 2014 Jul 23;11:126. doi: 10.1186/1742-2094-11-126 (PMC4128581; doi:10.1186/1742-2094-11-126)
Supplement: Additional file 2 — Histone H3 methylation pattern in R1 and P8 tissues. [file 1742-2094-11-126-S2.pdf]

Supplementary S3. Histone H3 methylation pattern in R1 and P8 tissues.

(A) Mono-, di- and tri-methylation in lysine 4 histone H3 (H3K4me, H3K4me2, H3K4me3) in splenocytes from 9 month-old P8 and R1 mice were determined by ELISA (n=3/group); trimethylated histone H3 lysine 4 (H3K4me3) was determined by Western blots in cortex (B) and hippocampus (C) from 9 month-old SAMP8 and SAMR1 mice and quantified by scanning densitometry (n=3/group and n=6/group, respectively). H3K4me3 was corrected by total histone H3 and data was represented as mean ± SE; t-test for independent samples results are indicated as * $p<0.05$.

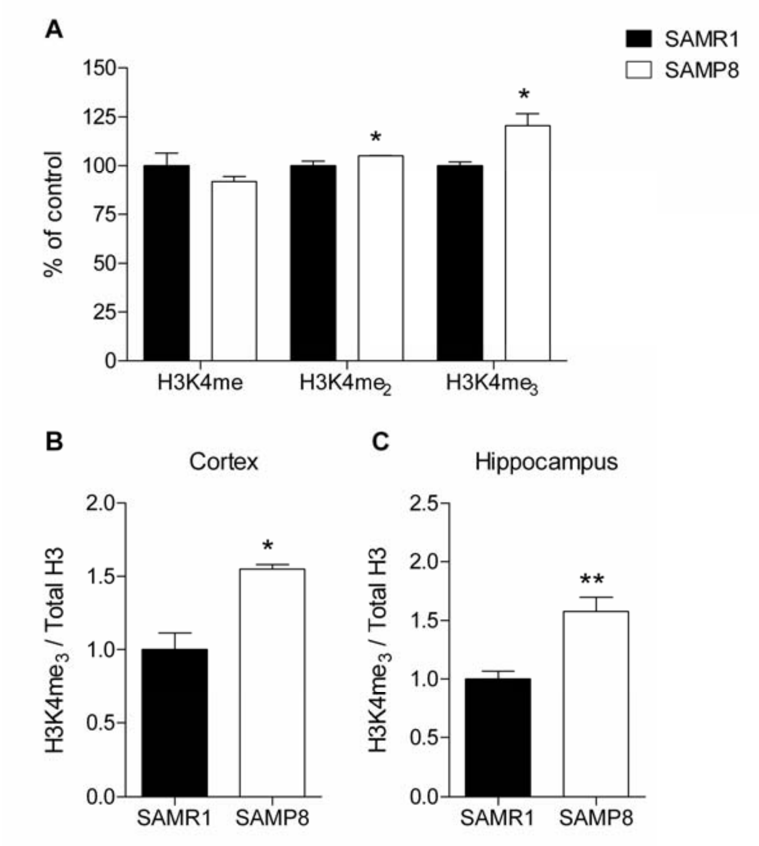

Materials and Methods

Histone proteins were extracted from splenocytes using EpiQuik Total Histone Extraction kit (Epigentek) and quantification of methylated H3K4 was performed using the EpiQuik Global Pan-Methyl Histone H3-K4 Quantification Kit (Epigentek), in accordance with the manufacturer's protocol. Data were normalized to SAMR1 levels.

Acidic histone fractions from cortices and hippocampi were obtained as described by Fischer et al. (2007). Briefly, tissues for immunoblotting were lysed in a buffer containing 50 mM Tris HCl, 150 mM NaCl, 2 mM EDTA, protease inhibitor cocktail (Pierce) and 1% Triton-X100 at 4°C for 15 min and then centrifuged 10 min at 400 x g. The remaining pellets were dissolved in the same buffer supplemented with 0.2 M HCl and incubated on ice for 30 min. After a centrifugation, the supernatants containing the histone fractions were immediately frozen at -80°C.

Histone fractions (5µg) were electrophoretically analyzed on 12% bis-Tris polyacrylamide gels and transferred to a 0.45µm PVDF membrane. Membranes were blocked with 5% BSA in PBS for 1 h and incubated overnight at 4°C with the specific primary antibodies (1:1000, Millipore). Membranes were washed and incubated with peroxidase-labeled secondary antibodies for 1 h at room temperature. Immunoreactive bands were detected by autoradiography. Specific bands from Western blot were quantified by scanning densitometry using Quantity One® 1-D analysis 4.6.3 software (Bio-Rad USA, Life Science Research, Hercules, CA). Histone modifications levels were corrected by total histone 3 expression.
