# Supplementary material for: Rcor2 underexpression in senescent mice: a target for inflammaging?
Source: J Neuroinflammation. 2014 Jul 23;11:126. doi: 10.1186/1742-2094-11-126 (PMC4128581; doi:10.1186/1742-2094-11-126)
Supplement: Additional file 3 — Methyltranferase inhibitor MTA reduces lipopolysaccharide (LPS)-induced Il6 gene expression in C6 glioma cells. [file 1742-2094-11-126-S3.pdf]

Supplementary S4. Methyltranferase inhibitor MTA reduces LPS–induced Il6 gene expression in C6 glioma cells

C6 glioma cells were treated for 1 h with LPS (1µg/ml) in C6 in the absence or presence of methylthioadenosine (MTA) (1mM) (n=6/group). Gene expression levels were determined by real time PCR. Mean ± standard error are represented. Pairwise comparisons with LSD adjustment from two-way ANOVA are indicated as ** p<0.01; *** p<0.001.

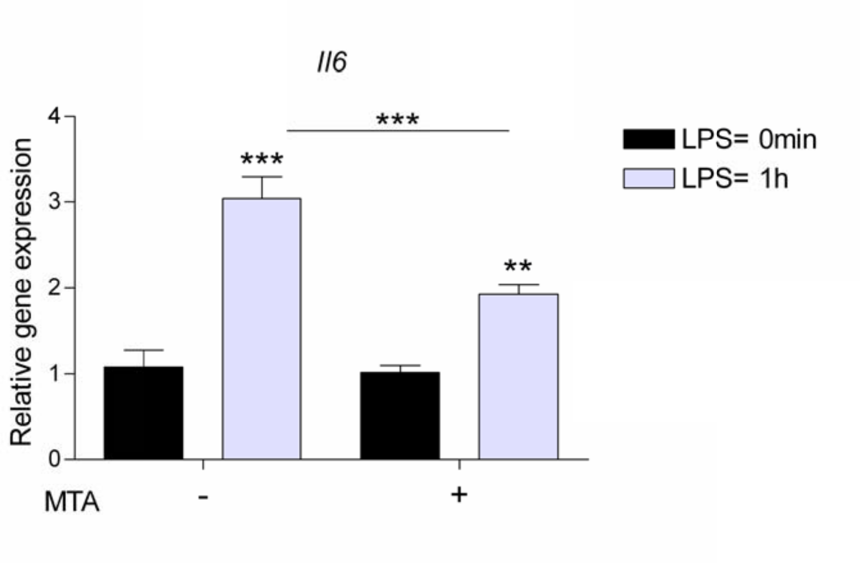

Materials and Methods

Rat C6 glioma cells (purchased from the European Collection of Cell Cultures, ECACC) were cultured as monolayer and grown in F12/DMEM medium (Gibco-Invitrogen) supplemented with 10% foetal bovine serum (Invitrogen), 1% non essential a/a's (Gibco-Invitrogen), 1/500 gentamicine (Gibco-Invitrogen) and maintained at 37°C and 5% CO_2 in a humidified environment.

For each treatment, C6 cells were seeded in a six-well plate and cultured in F12/DMEM without serum for 12 h prior to stimulation with LPS (1µg/ml) for different time periods ranging from 30 min to 6 h and exposed to MTA (1mM) or vehicle during LPS treatment when indicated.
