# Supplementary material for: Rcor2 underexpression in senescent mice: a target for inflammaging?
Source: J Neuroinflammation. 2014 Jul 23;11:126. doi: 10.1186/1742-2094-11-126 (PMC4128581; doi:10.1186/1742-2094-11-126)
Supplement: Additional file 4 — Pro‒inflammatory gene expression response in P8 hippocampus after intraperitoneal lipopolysaccharide (LPS) injection. [file 1742-2094-11-126-S4.pdf]

Supplementary S2. Pro-inflammatory gene expression response in P8 hippocampus after intraperitoneal LPS injection.

(A) *Rcor2*, (B) interleukin 1 beta, (C) tnf-alpha and (D) interleukin 6 gene expression levels in hippocampus from 12 month-old R1 after intraperitoneal LPS injection (n=3-4/group). Real time PCR was performed and mean ± standard error are represented. Two-way ANOVA results are indicated as * $p<0.05$.

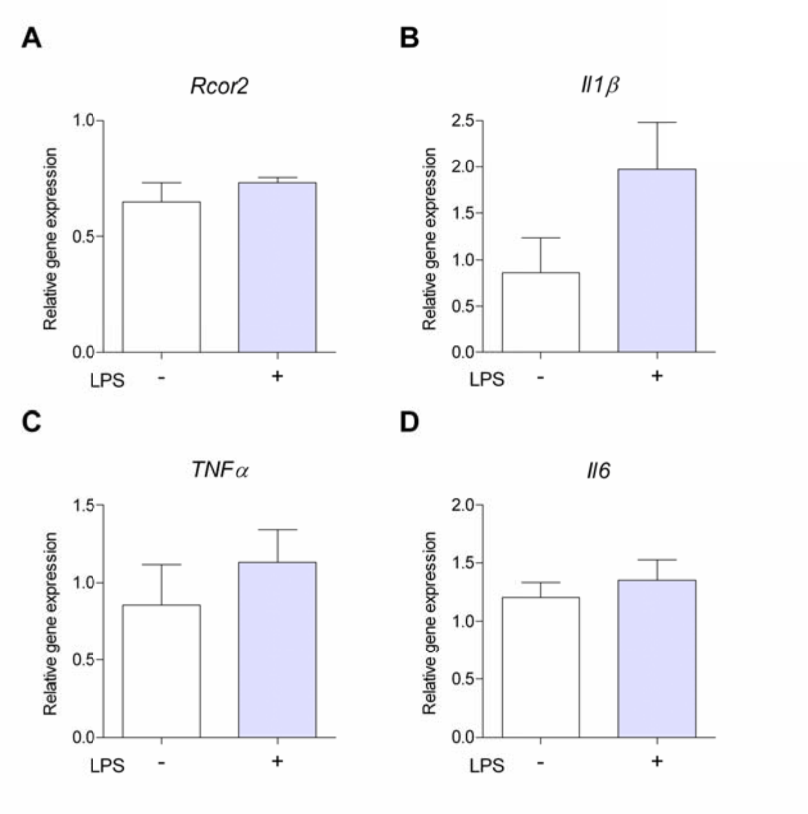

Materials and Methods

SAMP8 mice (males, 12 month-old, n=3/group) received an intraperitoneal (IP) injection of LPS (2.9 mg/kg; Sigma-Aldrich, St. Louis,Mo., USA), or an equal volume of saline. Three hours after treatment, blood samples were collected and mice were sacrificed by cervical dislocation. Tissues were collected and immediately frozen in liquid nitrogen and stored at -80°C until processed.

Detection of TNF-α, IL1-ß and IL6 in plasma were performed by enzyme-linked immunosorbent assay, in accordance with the protocol provided by the manufacturer (Mediclinics, R&D Systems and BioNovarespectively).
